# Drug Trafficking Organizations and Local Economic Activity in Mexico

**DOI:** 10.1371/journal.pone.0137319

**Published:** 2015-09-08

**Authors:** Felipe González

**Affiliations:** Department of Economics, University of California, Berkeley, California, United States of America; Beihang University, CHINA

## Abstract

Little is known about the relationship between illegal firms and local economic activity. In this paper I study changes in satellite night lights across Mexican municipalities after the arrival of large drug trafficking organizations in the period 2000–2010. After accounting for state trends and differences in political regimes, results indicate no significant change in night lights after the arrival of these illegal firms. Estimated coefficients are precise, robust, and similar across different drug trafficking organizations.

## Introduction

Informal and illegal economic activities are spread across the world and represent a large share of economic activity. This is particularly true in Latin America, where approximately 50 percent of the labor force works in unregulated markets [1]. Although understanding this side of the economy is important for many reasons, there is a limited amount of work due to data constraints. The availability of new data can help us fill this gap.

In this paper I study one of the largest illegal economic activities in Latin America: drug trafficking in Mexico. The supply side of this market is composed by big strategic profit-maximizing firms that have spread across Mexico for the last twenty years [2]. Under the Presidency of Felipe Calderón (2007–2012) the Mexican government has made an effort to stop their operations using crackdowns. As a result, more than 50,000 people have died in the last six years and drug traffic has not declined [3].

Understanding the behavior of these (and other) illegal organizations is crucial to designing better public policy to control drug production and trafficking, one of the largest challenges faced by the region. In this paper I estimate the effect of drug trafficking organizations on local economic activity in Mexico. The main objective is to understand how movements of illegal firms across space and time affect local economic activity.

The paper is divided into two parts. In the first part I describe drug trafficking organizations, their location across space, and their expansion over time. In the second part I exploit changes in the location of drug trafficking organizations across Mexico’s municipalities for each year between 2000 and 2010 to estimate their effect on local economic activity, measured as satellite night lights. My empirical analysis accounts for specific state trends and differences between the governments of Vicente Fox (2000–2006) and Felipe Calderón (2007–2012). Main results indicate no significant changes in a municipality’s night lights after the arrival of a drug trafficking organization. This result is precise and robust to different measures of both dependent and independent variables.

## Drug Trafficking Organizations in Mexico

### Industry

Drug trafficking organizations (DTOs) in Mexico are illegal firms involved in drug production, trafficking, and other criminal activities [4]. The number of DTOs operating in Mexico has been increasing over time as a result of fractionalization [5]. Although alliances exist across DTOs, many of them are rivals competing for revenues. It has been estimated that earnings in the drug industry are somewhere between 14 and 48 billion US dollars annually (U.S. State Department, 2009).

The global drug industry is complex as there are many types of drugs being produced, and consumers and producers are had to observe. For example, the vast majority of cocaine is produced in Bolivia, Peru and Colombia but consumed elsewhere [6]. When producers and consumers are located in different parts of the world, trafficking becomes an important part of the supply chain. Mexico is part of the production, trafficking, and consumption sides of the global drug industry.

**Fig 1:**
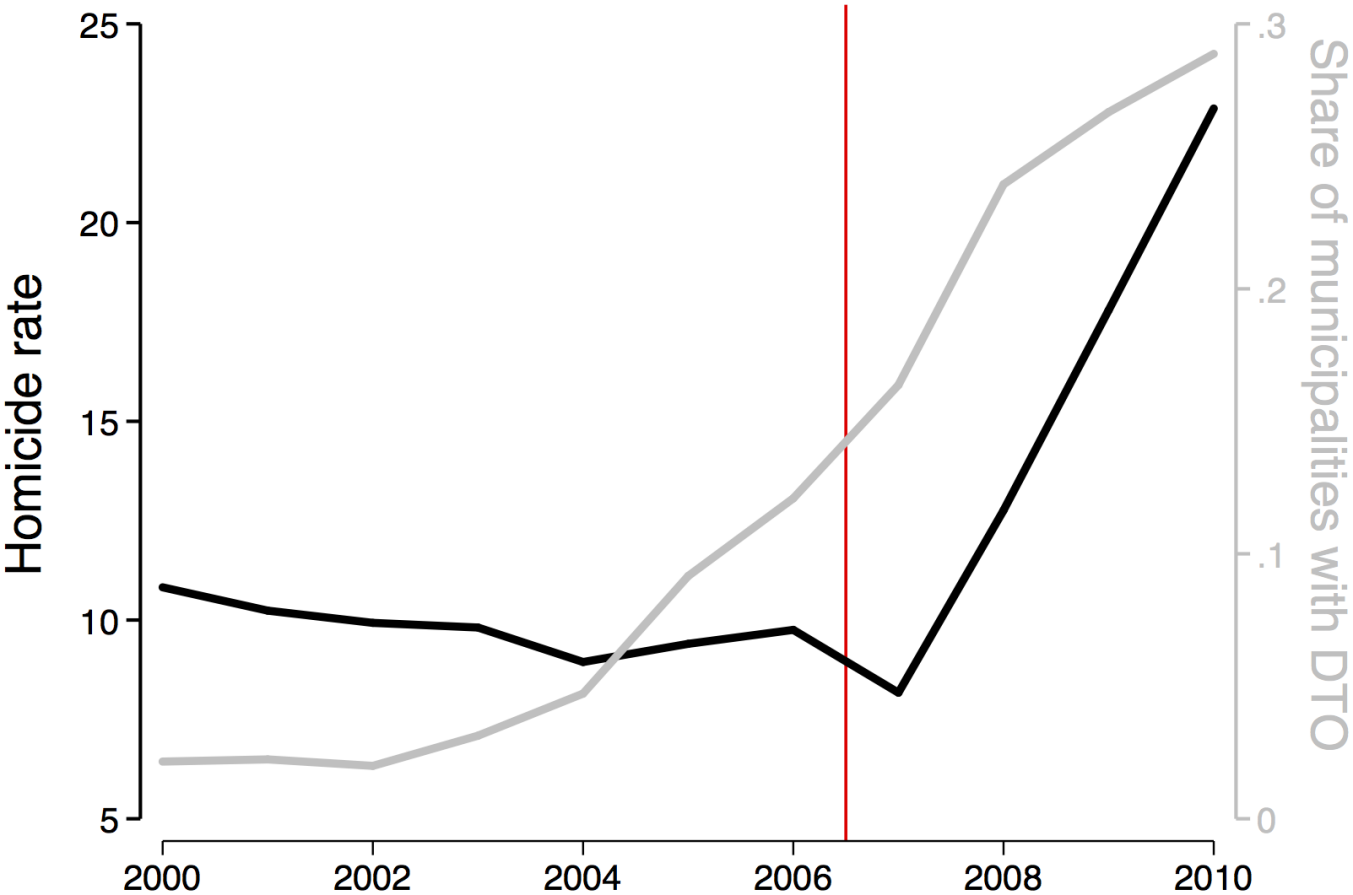
Different periods. The left axis of this figure presents homicides per 100,000 inhabitants in Mexico. The right axis presents the number of municipalities with at least one DTO over the total number of municipalities in Mexico. The red vertical line marks the beginning of Felipe Calderon’s government (December 2006—November 2012).

Although the Mexican government has been fighting the drug industry in the last decades, DTOs have been expanding across space and violence has increased dramatically (see Fig 1). The movements of these illegal firms across space has been associated to increases in violence as DTOs fight to control strategic territories [3]. We know little, however, about how local economic activity changes after the arrival of these large illegal firms.

### Network analysis

Using data on the geographic location of DTOs in Mexico, originally constructed by [7], I can provide some observable differences across organizations. In particular, I combine the observed geographic location of each DTO at the municipality level in 2010 with a network analysis to provide new descriptive statistics about drug operations. In particular, I use [8] least cost path to estimate trafficking routes from municipalities where DTOs operate to each U.S. entry point. I consider the simplest version of the algorithm: travel costs are a linear function of the distance traveled through the road network from the origin to the destination.

**Fig 2:**
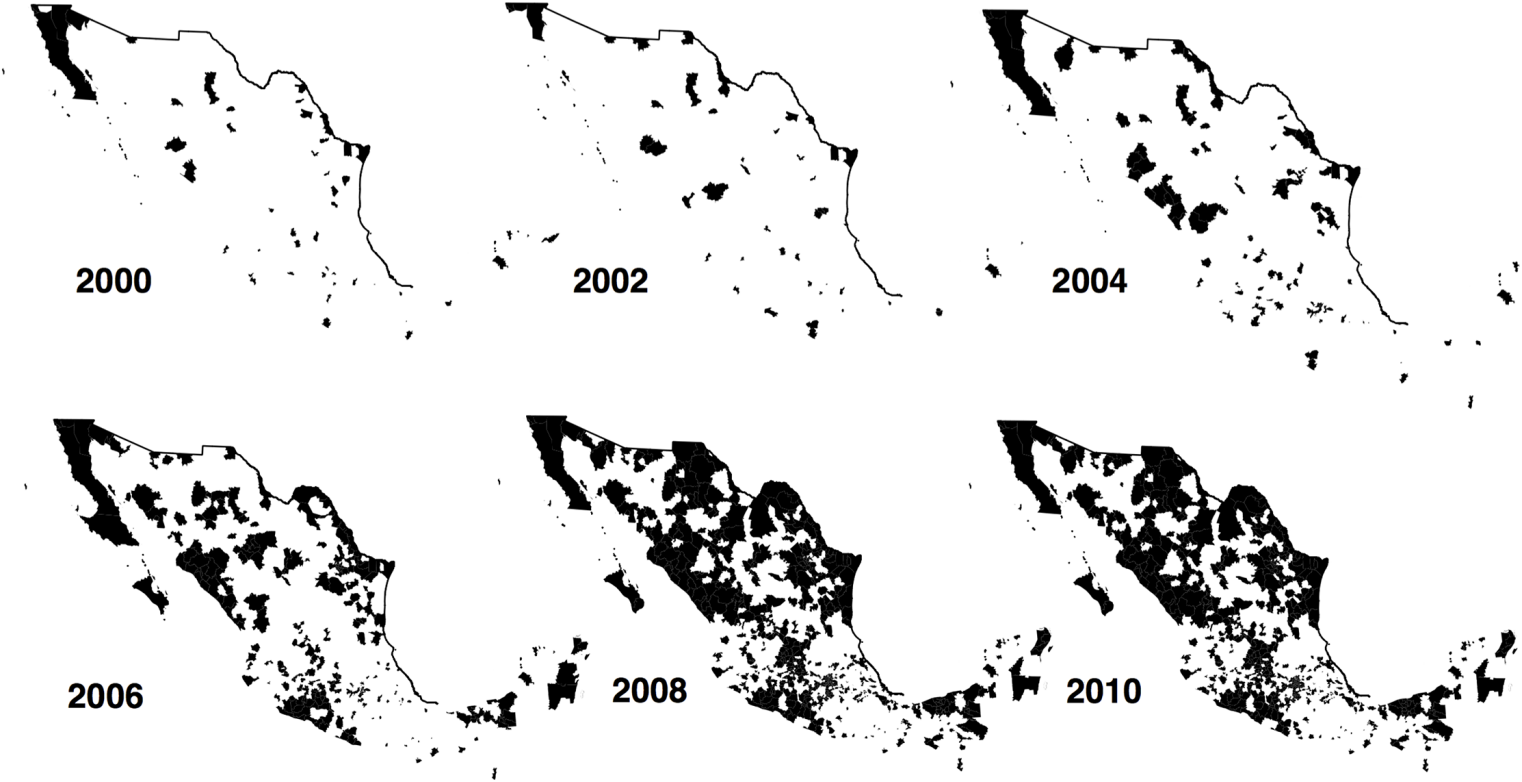
Drug trafficking organizations across space and time. Municipalities colored in black have at least one DTO operating. Data from [7].

There were ten different trafficking organizations actively operating in 2010. Fig 2 presents their geographic presence across Mexican municipalities every two years in the period 2000–2010. In order to estimate which U.S. entry point each DTO uses to traffic drugs, I perform the following steps empirically.

**Fig 3:**
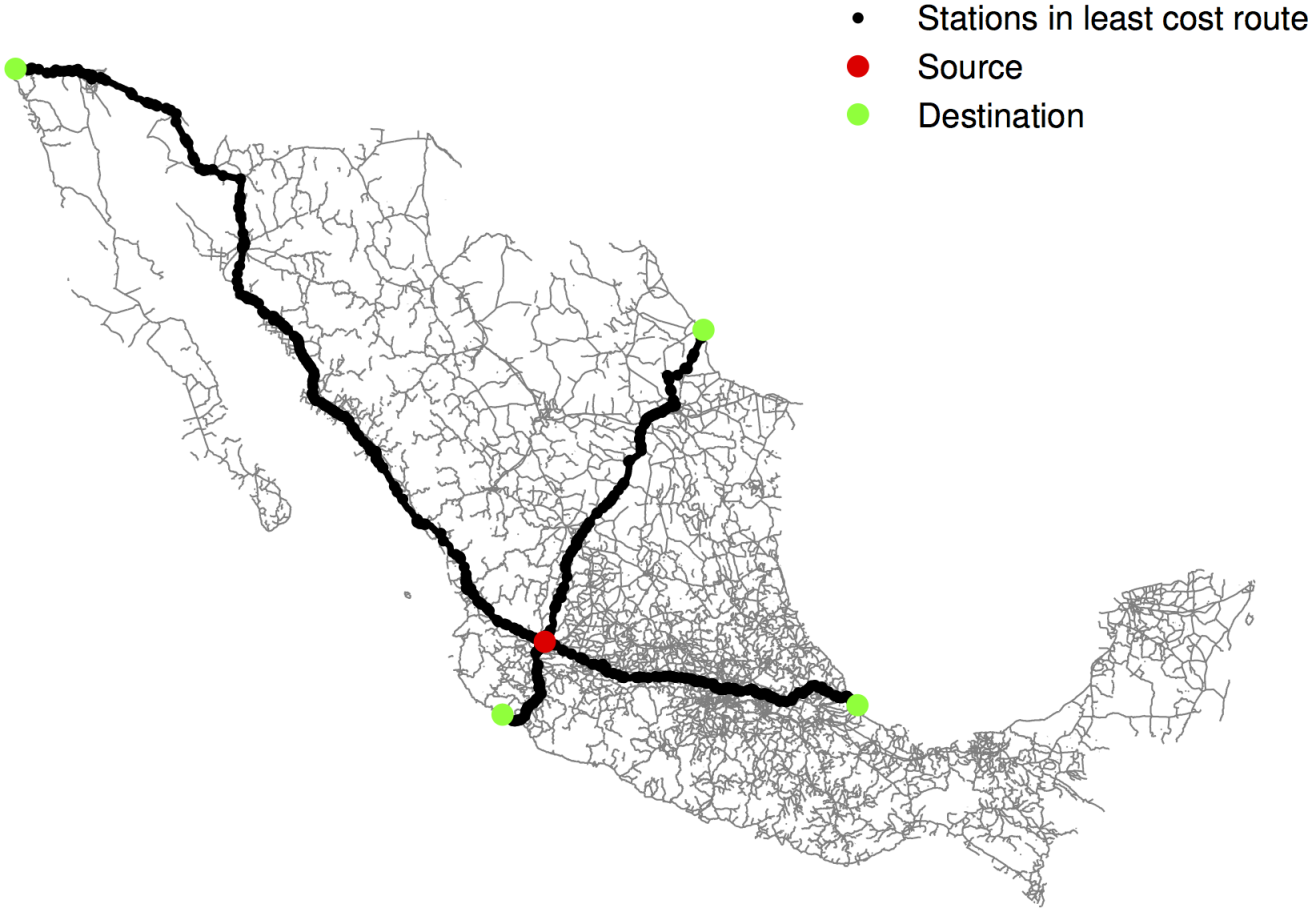
Road network. Mexico’s road network and an example of Dijkstra’s least cost route algorithm.

1. Assume that each DTO is operating at the municipality’s centroid. Then, look for the closest point (in an Euclidean distance sense) in the road network, and consider that point as the origin.
2. Calculate the distance through the road network to all U.S. entry points using Dijkstra’s algorithm over the road network in Mexico. An example is depicted in Fig 3, where 4 of the 27 U.S. entry points in the analysis are highlighted. There could be other entry points used by DTOs (e.g., small airports). In this sense, this exercise should be interpreted with caution. This procedure gives us a (*N*_*i*_ × *E*) matrix that I call 𝓓, where *N*_*i*_ is the number of municipalities where DTO *i* is active and *E* is the number of potential destinations.
3. Assume that each DTO is trafficking drugs through the closest U.S. entry point, which collapses the matrix 𝓓 into an (*N* × 1) vector I call 𝓒. The vector 𝓒_*i*_ represents the empirical distribution of distances for each DTO from places of operations to the closest U.S. entry points.

**Fig 4:**
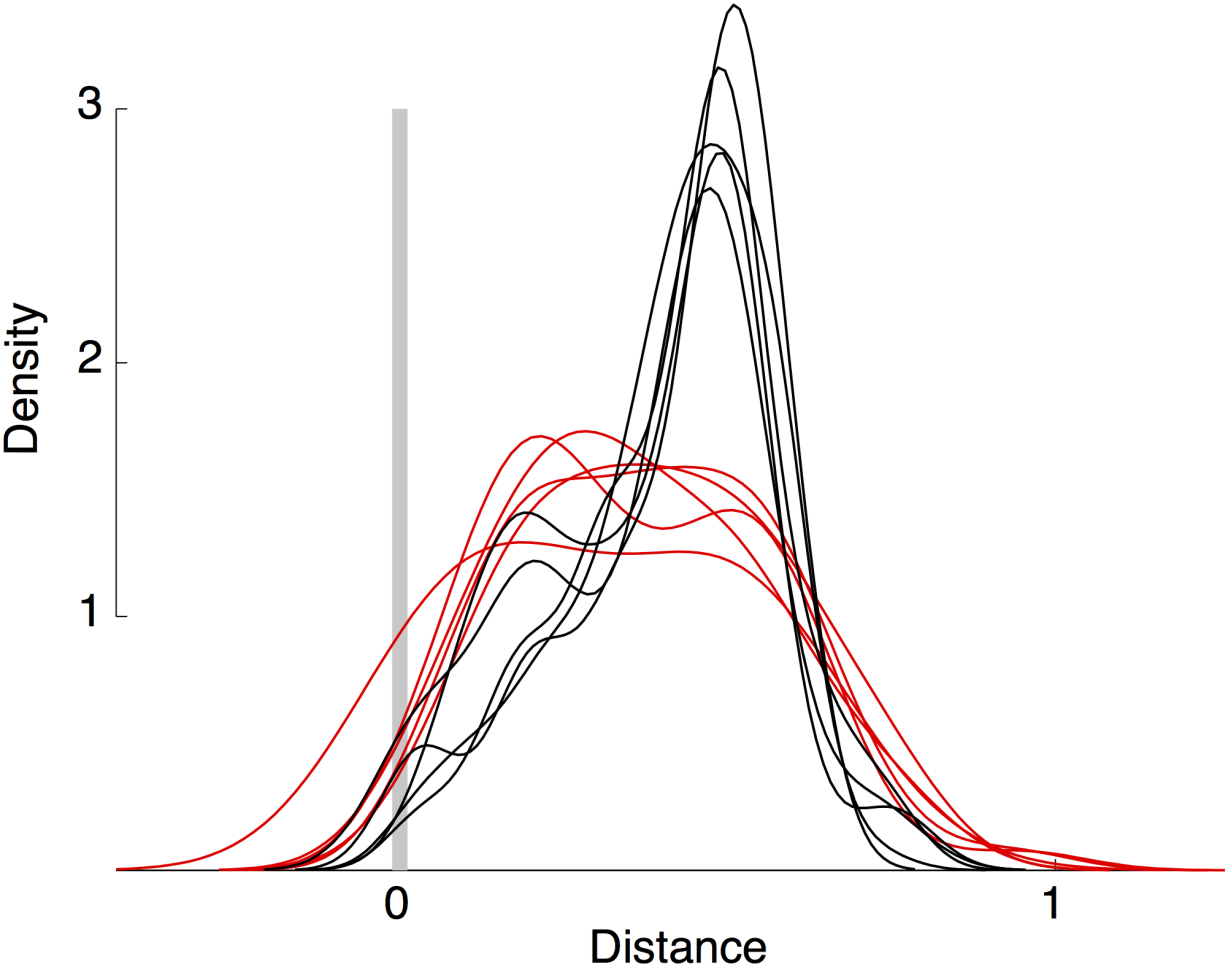
Distance to U.S. entry points. Estimated distances from places of operation to U.S. entry points using Dijkstra’s algorithm over the road network in Mexico. Kernel density estimations using an Epanechnikov kernel and the default optimal bandwidth.

The (normalized) empirical distribution of distances for each DTO is presented in Fig 4. There are ten densities, each one corresponding to a different DTO. Remarkably, there seems to be (at least) two underlying data generating processes. This suggests there are different types of DTOs: one group operates relatively far from destination points (black), and another group operate relatively close to destination points (red). In the following section I explore the heterogeneous association between DTO presence and local economic activity across these two groups.

## Data and Methods

In this section I describe the data and methods to estimate the effect of DTO presence on local economic activity. By combining annual remote sensing data to measure local economic activity and annual DTO presence for the period 2000–2010 I can statistically test how the arrival of a DTO affects economic activity in a regression framework analysis.

### Data

As previously mentioned, DTO presence at the municipality-year level was originally constructed by [7]. The authors track the location of DTOs annually between 1990 and 2010 using an algorithm to filter information from Google News. In order to understand the time variation in this dataset, in Fig 1 I have plotted the share of all municipalities in which there is at least one DTO operating. We can clearly see that after 2004 there is a remarkably expansion of DTOs across municipalities in Mexico. Before the year 2000, however, DTOs are located in a small set of municipalities. As there is little variation in DTO presence across municipalities in the period 1990–2000, the following analysis focuses on the period 2000–2010. Fig 2 presents the geographic dispersion of DTOs during this period.

Measuring economic activity at the municipality level every year is difficult. However, recent research suggests it is possible. A number of articles have shown that we can measure economic activity at the local level using satellite night lights [9, 10, 11, 12]. Although satellite night lights as seen from outer space are incapable of distinguishing between legal and illegal economic production, we can easily use them to test whether the arrival of a DTO at some municipality is correlated with an increase (or decrease) in total local economic activity.

Satellite night lights data was made publicly available by [13]. Satellites that collect these data orbit the Earth every day between 20:30 and 22:00, sending images of every location between 65 degrees south latitude and 65 degrees north longitude at a resolution of 20 arc seconds (≈ 1 km at the equator). The data used in this study corresponds to “stable lights” collected by satellites F15 and F16. The unit of observation is a pixel-year and is available for the period 1992–2013. For the purposes of this study the pixel-year data has been aggregated at the municipality-year level.

In addition to incorporating variation in both legal and illegal economic activity, satellite night lights also provide higher frequency variation in economic production than standard census data, only available in Mexico every five years. As DTOs are constantly moving across municipalities from year to year, satellite night lights provide an ideal way to measure their effect on the local economy. As [10] states: “Lights growth gives a very useful proxy for GDP growth over the long term and also tracks short-term fluctuations in growth”.

### Methods

Econometrically, I exploit annual variation in both DTO presence and night lights using panel data methods. This strategy has the advantage of using *within* municipality variation to identify the parameter of interest. This means that each municipality is being compared to itself when it hosted and when it did not host a DTO. In particular, I run regressions of the following form:

$$\begin{array}{c} \ell_{mst}=\beta \cdot {\text{DTO}}_{mst}+\xi_{m}+\zeta_{t}+\varepsilon_{mst} \end{array}$$ (1)

$$\begin{array}{c} \ell_{mst}=\tilde{\beta }\cdot {\text{DTO}}_{mst}+{\tilde{\xi }}_{m}+{\tilde{\zeta }}_{t}+\gamma_{st}+{\tilde{\varepsilon }}_{mst} \end{array}$$ (2)

where *ℓ*_*mst*_ is a night lights variable in municipality *m*, state *s*, and year *t*, *ξ*_*m*_ is a full set of municipalities fixed effects, *ζ*_*t*_ is a full set of year fixed effects, DTO_*mst*_ is an indicator variable that takes the value of one if a DTO is active in municipality *m*, state *s*, and year *t*, *γ*_*st*_ is a state-year fixed effect, which removes variation in any differences in lights sensitivity across satellites and changes in worldwide economic conditions, and *ε*_*mst*_ is an error term clustered at the municipality level. All dependent variables have been normalized between zero and one to facilitate interpretation of coefficients.

I present results from two specifications, Eqs (1) and (2), where the main difference is the inclusion of *γ*_*st*_. Eq (2), however, is the preferred specification as it accounts for state trends in the period under study. In addition I present results separately for the periods 2000—2006 and 2007–2010 to account for differences across political periods.

Notice that *β*, the parameter of interest, is the causal effect of DTO on economic activity under the assumption that legal and illegal economic activity are uncorrelated. As this is unlikely to be the case for many reasons, I interpret estimates of *β* as partial correlations. One reason to believe *β* might represent the causal effect of DTO on economic activity is that illegal activities change in a higher time-frequency than legal activities. Another reason is to believe DTOs move to places independently of their legal economic activities, but they have an effect on them, in which case *β* represents the reduced form between DTO and economic activity. In an omitted variable bias scenario, a positive correlation between legal and illegal economic activity biases *β* upwards, and a negative correlation biases it downwards. Although is hard to ex ante know which is more likely, there is evidence of the latter [14].

## Results

Table 1-A present estimates of Eqs (1) and (2) using all municipalities in Mexico observed yearly in the period 2000–2010. Columns 1 and 2 present estimates using the average of satellite night lights in each municipality-year as the dependent variable. In column 1 we observe that the presence of a DTO increases economic activity by 0.25 standard deviations, and this is statistically significant at the 1% level. In column 2 I include a full set of state-year fixed effects to control for specific state trends and to account for crackdowns that occurred during Felipe Calderon’s government. The inclusion, in a very flexible way, of this state-year fixed effects does not change the pattern, although the effect is somewhat smaller (approximately 0.08 standard deviations).

**Table 1:** Drug trafficking organizations and local economic activity.^†^

*Dependent variable:*	*Average Night Lights*		*Per Capita Night Lights*	
	(1)	(2)	(3)	(4)
		**A. Period 2000–2010**		
DTO presence	0.25***	0.08***	0.24***	0.07***
	(0.02)	(0.02)	(0.02)	(0.02)
Observations	26,950	26,950	26,983	26,983
		**B. Period 2000–2006**		
DTO presence	0.10***	0.01	0.09***	0.00
	(0.03)	(0.03)	(0.03)	(0.03)
Observations	17,150	17,150	17,171	17,171
		**C. Period 2007–2010**		
DTO presence	0.03	0.03	0.02	0.02
	(0.03)	(0.03)	(0.03)	(0.03)
Observations	9,800	9,800	9,812	9,812
Municipality F.E.	Yes	Yes	Yes	Yes
Year F.E.	Yes	Yes	Yes	Yes
State-Year F.E.	No	Yes	No	Yes

^†^ Robust standard errors clustered at the municipality level are presented in parentheses.

Significance level: *** *p* < 0.01. All dependent variables have been normalized between 0 and 1 to facilitate interpretation of coefficients. Night lights data takes a value between 0 and 63 for each approximately (1-km) pixel. Pixel data was aggregated at the municipality level using the zonal statistics package in QGIS in each year. Population data is from the 2010 Mexican Census. DTO presence (number) is a dummy that equals to 1 if a DTO is active in a municipality-year (the sum of presence across DTOs in a municipality-year).

The following two columns (3 and 4) repeat the same exercise but change the way we measure local economic activity (*ℓ*_*mst*_). In this case *ℓ*_*mst*_ is per capita night lights. Results are essentially unchanged and I cannot reject that coefficients in columns 3 and 4 are statistically different from coefficients in columns 1 and 2. These and all following results are similar if I replace DTO presence by the number of DTOs operating in a municipality-year.

Perhaps surprisingly, these results suggest that DTO presence is associated with a somehow small *increase* in local economic activity, Nevertheless, the possibility of an omitted variable correlated with both DTO presence and local economic activities remains. To improve our understanding of this empirical association between DTO presence and satellite night lights, I now examine its heterogeneity across different periods of time. One might worry that the association I have documented is driven by differences in policy platforms across different governments. This is particularly important in the case of Mexico because of the different policies implemented by Vicente Fox (2000–2006) and Felipe Calderón (2006–2010) to fight DTOs. If this is the case, the average association between these two variables could be masking heterogeneity across political periods.

In Table 1-B and 1-C I present estimates of Eqs (1) and (2) for the two political periods between 2000 and 2010. Interestingly, the effect is now considerably smaller, with no significant relationship between DTO presence and satellite night lights in the regression specification with state-specific trends. When compared with results in Table 1-A, this suggest that the positive association between DTO and night lights over the period 2000–2010 is driven by differences across political regimes.

A fully flexible way to estimate the empirical association between DTO presence and night lights is to calculate it for every year in the period under study. This can be done by estimating the following regression equation:

$$\begin{array}{ccc} \ell_{mst} & = & \beta_{t}\cdot \left({\text{DTO}}_{mst}\times T_{t}\right)+\xi_{m}+\zeta_{t}+\gamma_{st}+\varepsilon_{mst} \end{array}$$ (3)

where ***T***_*t*_ = (*T*_2000_ … *T*_2010_) is a matrix composed by indicators that takes the value of one for year *t* and zero otherwise, and ***β***_*t*_ = (*β*_2000_ … *β*_2010_) is a matrix with the coefficients of interest. Note that a changing association between DTO presence and night lights translates into differences in *β*_2000_, …, *β*_2010_. In addition, note that the estimated coefficient $\hat{\beta }$ in Table 1-A is simply a weighted average of the coefficients ${\hat{\beta }}_{2000},\ldots ,{\hat{\beta }}_{2010}$

**Fig 5:**
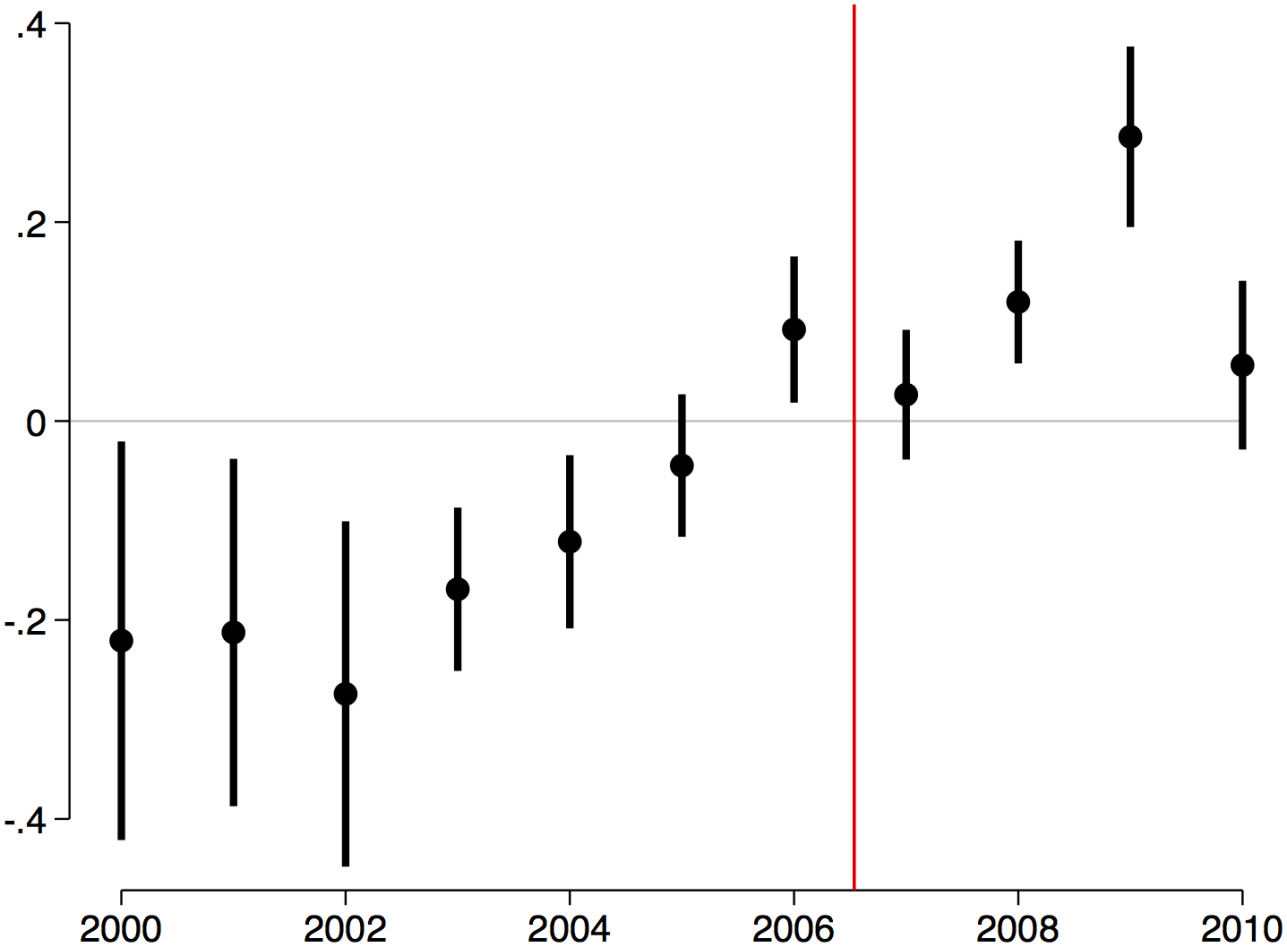
DTO presence and night lights over time. This figure presents estimates of ${\text{β}}_{t}=({\hat{\beta }}_{2000}\;\ldots \;{\hat{\beta }}_{2010}$) from the following regression: *ℓ*_*mst*_ = ***β***_*t*_⋅(DTO_*mst*_×***T***_*t*_)+*ξ*_*m*_+*ζ*_*t*_+*γ*_*st*_+*ε*_*mst*_, where *ℓ*_*mst*_ is night lights in municipality *m*, state *s*, and year *t*, *ξ*_*m*_ is a full set of municipalities fixed effects, *ζ*_*t*_ is a full set of year fixed effects, DTO_*mst*_ is an indicator variable that takes the value of one if some DTO is active in municipality *m*, state *s*, and year *t*, *γ*_*st*_ is a state-year fixed effect, *ε*_*mst*_ is an error term clustered at the municipality level.

Fig 5 present results of estimating Eq (3). It is clear from this figure that there is substantial heterogeneity in the association between DTO presence and night lights across years. Before 2004 the arrival of a DTO to a municipality is associated with a decrease in local economic activity. After 2006, however, this association begins to be positive. Remarkably, this reversal in the association of interest coincides with the time in which DTOs began their expansion across the country.

Finally, following the network analysis in section, Table 2 presents estimates of Eq (1) in which the DTO presence indicator has been divided in two different variables. The first (second) variable takes the value of one in a municipality-year if there is a DTO that typically operates close to (far from) U.S. borders, and zero otherwise. I again present estimates for three periods of time using average night lights as dependent variable. Results indicate that there is no statistically significant association between DTO presence and local economic activity. Moreover, point estimates for the two types of DTOs are statistically similar.

**Table 2:** Heterogeneity by type of DTO.^‡^

*Dep. variable:*	*Average night lights*		
	(1)	(2)	(3)
DTO operating close to U.S. border (*α*)	0.08***	0.01	0.03
	(0.02)	(0.03)	(0.03)
DTO operating far from U.S. border (*β*)	0.06**	0.05	-0.03
	(0.03)	(0.05)	(0.03)
*H*_0_: *α* = *β* (*p*-value)	0.63	0.52	0.21
Municipality F.E.	Yes	Yes	Yes
Year F.E.	Yes	Yes	Yes
State-Year F.E.	Yes	Yes	Yes
Years	2000–2010	2000–2006	2007–2010
Observations	26,950	17,150	9,800

^‡^ Robust standard errors clustered at the municipality level are presented in parentheses.

Significance level: *** *p* < 0.01, ** *p* < 0.05. All dependent variables have been normalized between 0 and 1 to facilitate interpretation of coefficients. Night lights data takes a value between 0 and 63 for each approximately (1-km) pixel. Pixel data was aggregated at the municipality level using the zonal statistics package in QGIS in each year.

## Conclusion

Although illegal economic activity is difficult to measure, satellite night lights data captures both legal and illegal activity, thus providing an opportunity to measure the impact of illegal organizations on local economic activity. In this paper I have shown that there are no significant changes in satellite night lights after the arrival of a drug trafficking organization to a municipality in Mexico.

The availability of new and worldwide remote sensing datasets, together with computer algorithms that track illegal organizations across space and time, provide a unique opportunity to learn more about an important but understudied side of the economy: illegal markets. In addition, more research is needed to understand the interactions between the legal and illegal sectors of the economy.
